# Knowledge, attitudes, and practices of cardiopulmonary rehabilitation among physiotherapists in Lebanon

**DOI:** 10.1186/s43161-021-00060-w

**Published:** 2022-01-12

**Authors:** Rebecca Farah, Wim Groot, Milena Pavlova

**Affiliations:** 1 Department of Health Services Research, CAPHRI, Maastricht University Medical Center, Faculty of Health, Medicine and Life Sciences, Maastricht University, Maastricht, Netherlands; 2 Department of Rehabilitation and Physical Therapy (Group A), Delta-Chirec Hospitals Group, Brussels, Belgium

**Keywords:** Cardiopulmonary rehabilitation, Prevention, Physical activity, Lebanon

## Abstract

**Background:**

Insufficient physical activity is one of the leading mortality risks worldwide for cardiovascular and pulmonary diseases. Physiotherapists (PT) are core healthcare professionals who play a major role in the prevention of disease complications and in inspiring a healthy lifestyle. To identify challenges in the promotion of cardiopulmonary rehabilitation (CR) in Lebanon, a survey was conducted among PT and physiotherapy students. The aim was to assess the knowledge, attitudes, and practices of CR in Lebanon.

**Results:**

The response rate was 46.1% (*N* = 322). Results show that 24.5% of respondents have good to excellent knowledge about CR. More than 60% of the respondents indicate possible barriers to starting a CR program, and one of two respondents identify the absence of skills as a main barrier. Findings highlight the importance of the role of PT as a mediator to increase a healthy lifestyle among patients and to promote the prevention of cardiovascular diseases and pulmonary diseases in the country.

**Conclusions and recommendations:**

Our results support the evidence and clinical guidelines that PT play a major role by increasing the participation of patients in CR. A cost-effective CR program needs to be covered by the private and public system in Lebanon.

**Supplementary Information:**

The online version contains supplementary material available at 10.1186/s43161-021-00060-w.

## Background

Since the 1960s, cardiopulmonary rehabilitation (CR) has been the traditional mode for the delivery of prevention for patients suffering from cardiac diseases and chronic obstructive pulmonary diseases (COPD). Later on, an educational component aimed at educating patients about the importance of lowering risk factors, including smoking, diet, psychological well-being, has been added [1].

CR is more than exercise training for patients suffering from cardiopulmonary diseases. It includes all aspects of prevention. It emphasizes the prevention of disease complications and chronic disease–based strategies. Exercise is an important component of lifestyle risk factor management. This refers to cardioprotective therapy that is efficient and cost-effective according to international recommendations and guidelines based on evidence [2].

The 2019 Guidelines of the European Society of Cardiology and Prevention (ESCP) state that “exercise-based cardiac rehabilitation has consistently demonstrated its effectiveness in reducing cardiovascular mortality and hospitalizations compared with no exercise controls in patients with chronic artery diseases, and this benefit persists into the modern era Exercise has been referred to as a polypill due to its numerous beneficial effects on cardiovascular risk factors and system physiology” [2]. Exercise therapy is recognized as the “main component of secondary prevention” and CR. This is supported by systematic reviews and meta-analysis of RCTs, which urge that the exercise component of CR is essential, and better outcomes are achieved when this component is included (https://www.escardio.org/Guidelines/Consensus-and-Position-Papers/EAPC-Publications). Consequently, physiotherapists (PT) are playing an important role in the multidisciplinary team and have several prevention roles [1, 2].

In particular, PT have the knowledge and expertise to help in the promotion of well-being and a healthy lifestyle but also in the prevention of non-communicable diseases (NCDs) by encouraging their patients to adopt a healthy way of life by doing exercises even if it is not in their plan of treatment. PT enable patients to adapt to medical recommendations, to a specific environment, to meaningfully interpret health recommendations, to create targeted approaches to help individuals modify health behaviors, and to ensure that clinical and community services are integrated, available, and mutually reinforcing [3]. PT, as specialists in both exercise and health promotion, are in an ideal position to influence the health of the individual [4].

The PT roles include health education, direct interventions, research, advocacy, and collaborative consultation [5]. PT plays a major role in this according to the American Physical Therapy Association and World Confederation of Physical Therapy (WCPT) [3, 4]: first, in the management of the diseases and, second, in the overall health promotion of the population. PT need to support and treat patients suffering from cardiopulmonary diseases to be physically active as part of their treatment. This requires knowledge and skills to assess each person’s clinical condition. Education, know-how, and the transmission of knowledge in prevention should start at the university for physiotherapy students and then continue through their daily practice. To better educate physiotherapy students in the prevention role of cardiopulmonary rehabilitation, it is crucial that they, as future healthcare professionals, learn how to promote a healthy lifestyle among the patient to increase the awareness for prevention. In addition, physiotherapy students are involved in the physiotherapy practice from the beginning of their study. Specifically, during their training, physiotherapy students play an assisting role in the care for patients supervised by the PT.

In Lebanon, rehabilitation programs are still lacking due to an absence of this specialization in the university curriculum of PT students. A master degree in this field, either in general rehabilitation or in cardiovascular and pulmonary rehabilitation, does not exist and needs to be considered. For years, globally, attention has been given to the musculoskeletal and neurologic specialties in physiotherapy [6]. Approval for physical therapy sessions in case of cardiopulmonary conditions is complicated. The National Social Security Fund (NSSF) and health insurance companies are reluctant to reimburse the costs of it. The implementation of policies and procedures to take these rehabilitation programs into consideration are still absent in the majority of the medical establishments, even though these are essential to decrease the burden of cardiopulmonary diseases in the country.

The aim of the study is first to assess the knowledge, attitudes, and practices (KAP) among PT and physiotherapy students toward cardiopulmonary rehabilitation and second to identify barriers faced by the respondents and to investigate the role of PT in secondary prevention. It is relevant to include PT students in the study as future healthcare professionals. Evidence on their knowledge, attitudes, and practices related to PT could help to identify potential gaps in their curriculum.

## Methods

This study had a quantitative cross-sectional design and used a structured, self-administrated KAP questionnaire. The questionnaire was anonymous. The self-administrated questionnaire was designed based on the three components of the KAP framework of the WHO [7]. We added two components: one about barriers faced by PT and a second one focused on the role of PT in prevention programs.

The survey was conducted during the 5th International Scientific Days conference of the National Institute of Physiotherapy. The conference was titled “Evidence Based: Clinical Practice & Prevention” and was held in October 2019 in Lebanon. The participants were PT registered with the Order of Physiotherapists in Lebanon (OPTL) and physiotherapy students. Approval to conduct this survey was obtained from the President of the OPTL and from the ethical committee of the OPTL, and our study was approved by the FHML Research Ethics Committee at Maastricht University. This study was performed according to the Declaration of Helsinki, which involves ethical principles for medical research involving human subjects according to the countries’ regulations as well as the applicable international requirements of the ICH-GCP [8]. We provided information about the study to every participant prior to the collection. We asked every study participant to give consent to participate and for publication of study results.

The Lebanese attendees of the conference were invited to fill in this questionnaire. Both PT and physiotherapy students were eligible to take part in this study. As mentioned earlier, physiotherapy students are involved in the physiotherapy practice since during their trainings, they play an assisting role in the care for patients supervised by the PT. Therefore, we considered it relevant to include PT students as well. No selection of respondents was made in advance. We did not have exclusion criteria; every conference participant was eligible to take part in this study if he/she wished. The participants did not receive any incentive to take part in the study.

The questionnaire was distributed by hand by the OPTL board and the filled-in questionnaires were collected and put in a box by the organizers of the 3-day OPTL conference. In total, 322 out of 698 conference participants returned a filled-in questionnaire.

The questionnaire was validated by experts in the field and pretested by three PT in Lebanon, and the recommended adjustments were made. Additional file 1 shows the 61 questions asked to the PT and physiotherapy students. As shown in Additional file 1, the questionnaire was divided in 6 parts after we collected first the sociodemographic data of the responders:

- Part 1 covered questions on knowledge and attitude of PT about CR.
- Part 2 explored the practice of PT on CR.
- Part 3 assessed the attitude of the PT toward CR.
- Part 4 investigated the role playing by PT in prevention.
- Part 5 assessed barriers faced by PT about CR.
- Part 6 evaluated if PT support CR.

At the beginning of the questionnaire, the informed consent question was included. The wording of questions was pretested before the survey by 3 physiotherapists in Lebanon and was adjusted. The English version of the questionnaire was used in the survey.

After the data collection and screening, data entry, cleaning, and analysis were done by using the software package SPSS version 24. The analysis consisted of descriptive statistics, namely frequencies, means, median, standard deviation, and frequencies for each response variable. The degree of association between sociodemographic data of the respondents and responses to the KAP questions and questions on the PT’s role was analyzed using regression methods.

## Results

In total 698 people, 275 PT and 423 physiotherapy students participated in the conference. Of all conference participants, 322 filled in and returned the questionnaire presented in Additional file 1. The overall response rate was 46.1%. The sociodemographic characteristics of the respondents are presented in Figs. 1, 2, 3, 4, and 5 in Additional file 2. Overall, 59.3% of the respondents are less than 25 years old and 17.7% are between 36 and 45 years old. In total, 51.2% are male, and 45.3% of them are physiotherapy students. All tables with study results are represented in Additional file 3.

### Knowledge of CR among PT and physiotherapy students

On average, the respondents indicated that they had medium level of knowledge about CR (41.3%, *n* = 133). About one quarter of the respondents (24.5%, *n* = 79) stated that they had good to excellent knowledge of CR while 30.1% (*n* = 97) of them stated that they had poor to very poor knowledge of CR.

In addition, nearly half of the respondents stated that they had medium level of knowledge about the multidisciplinary content/components of CR (45%, *n* = 145), and about one quarter had good to excellent knowledge of this CR aspect (25.5%, *n* = 82). About one third of the respondents (27%, *n* = 87) stated that they had poor to very poor knowledge of the multidisciplinary CR components (details can be found in Additional file 3, Table 1).

The majority of the respondents specified that they had good adherence to health promotion guidelines despite the absence of such programs in the country. Knowledge on WHO and WCPT guidelines on prevention and healthy promotion of CR is stated to be good to excellent by nearly half of the respondents (45.9%, *n* = 148). In addition, 46% stated that they had a good adherence to healthy promotion.

### Attitude of PT and physiotherapy students toward CR

Our results also provide information on the attitude toward CR. Most of the respondents in our survey had a positive attitude toward CR. Only about one third, 35.7% (*n* = 115), stated that CR in Lebanon is effective and 95% (*n* = 306) believed that CR will have an added value for the country. In total, 95.7% (*n* = 308) of the respondents agreed that CR could improve quality of life and lifestyle of a stable patient post-surgery or suffering from cardiovascular and pulmonary diseases. In addition, 95.3% (*n* = 307) indicated that CR could change patient behavior post-surgery or suffering from cardiovascular diseases (CVDs). Further details can be found in Additional file 3, Table 2.

### CR practice among PT and physiotherapy students

Regarding the practice of CR, our results indicate the respondents’ views on what type of patients are supposed to be eligible to start a CR. Most of the respondents stated that patients suffering from NCDs (post cardiac event, cardiac surgery, COPD patients, or with pulmonary diseases) should be “eligible candidates” for CR. Further details on this issue can be found in Additional file 3, Table 3.1.

Regarding the difficulty for a physiotherapist to refer patients to CR, about 80% of respondents agreed that it would be difficult to refer patients to a CR in the country. On the question regarding who should take the initiative to initiate this kind of program in the country, close to three quarter of the respondents thought that they “themself as physio” needed to initiate CR and a bit less than 70% stated that physicians need to select and refer the patients. About 40% of the respondents indicated that the insurance companies also play a role to initiate a CR program. Nearly half of the respondents believed that policy makers (person who established the policy) should play an important role to integrate the patient into a CR program (see Additional file 3, Table 3.2).

Additional analysis showed that almost no activity related to CR treatment was performed by PT at their work place or patient home during the preceding month. Specifically, more than 80% of the respondents did not treat patients suffering from cardiopulmonary diseases at their work place, private office or hospital setting during the preceding month. Also, more than 80% of the respondents did not treat patients suffering from cardiopulmonary diseases at patients’ home. Only “1 to 2 patients suffering from cardiopulmonary diseases” were treated at hospital or private clinic according to 63% (*n* = 11) of the PT in our study (see Additional file 3, Table 3.3).

### Barriers facing PT and physiotherapy students related to CR

Our survey provided data on whether the PT face barriers to integrate a CR program in Lebanon as well as the types of barriers. More than 60% of the respondents indicate possible barriers when patients are referred from physicians or primary care providers to start rehabilitation treatment. One of two respondents think that the main barrier is the absence of skills for implementing a CR program. The second most-supported barrier is the lack of specialists in this field. The third top barrier is the absence of specialized centers in Lebanon. Slightly more than 20% of the respondents indicated that a barrier is the lack of patients’ interest in CR when patients are referred from physicians to start a program. In addition, more than three quarters of the respondents think that a CR program will not benefit the patient and would not change patient behavior (details can be found in Additional file 3, Table 4).

In addition, more than half of the respondents confirmed that they will face barriers regarding the price of the program when patients are referred by primary health care providers to start treatment and think that not covering the program by insurance and NSSF (social security) is a barrier to enter to the CR program. No enough endorsement by physicians to start a CR program is also seen as a top barrier by more than half of the respondents. In addition, one quarter of the participants suggest additional barriers in the category “others” like the need of more education in this field of practice and more training for physiotherapy students, as well as to raise awareness about the CR importance and also the need to implement a master degree in this field practice which is missing in the country.

### Role played by PT in prevention through CR

Our respondents suggest that PT are playing a key role in the prevention of NCDs. More than 90% of the respondents supported this statement. Respondents agree that the PT have also other key prevention roles, such as to discuss the benefits of a healthy and active lifestyle with their patients, to encourage physical activity in their clinical practice on a daily basis (beyond therapeutic exercises) to promote the prevention of cardiovascular and pulmonary diseases. Findings show that the majority of PT are confident when giving advice to patients on physical therapy and act as a model for their patients. Regarding CR programs, PT are interested to participate in the assessment of patient’s body mass index (BMI) and to assess exercise capacity via 6-min walk test, make screening of cardiovascular risk factors, to give patient education, diet, and smoking cessation counselling. Finally, PT are playing a major role in psychological management during the patient treatment (see Additional file 3, Table 5).

### Support for types of CR delivery and recommendations by PT and physiotherapy students

Our findings suggest that PT and physiotherapy students in Lebanon support all kinds of CR programs in the country, namely inpatient and outpatient clinical setting as well as telemedicine delivery (details can be found in Additional file 3, Table 6). The majority of the respondents support the implementation of a CR center in the country as inpatient or outpatient clinics as well as future implementation of telerehabilitation.

PT and physiotherapy students in our study also stated several recommendations for CR in Lebanon. Specifically, the participants recommended more training and education in CR for physicians, to raise awareness about the CR importance in the country. In addition, they pointed out the need to solve the financial issue of covering the costs of patients’ enrollment in CR. They also recommended the promotion of CR for the prevention of NCDs and removing barriers to the enrollment of patients into CR.

### Associations

In addition, the degree of association between sociodemographic data of the respondents and responses to the KAP questions was analyzed using regression methods. However, we did not find any consistent pattern.

## Discussion

The aim of this first national study was to assess the KAP of PT and physiotherapy students toward CR in Lebanon and barriers faced by these groups in their CR practice. The role played by PT in the promotion of CR and prevention of NCDs was also considered. We focus the discussion on those results that are relevant for the PT practice in Lebanon.

Our results meet our expectations and are supported by other studies conducted in this field. In particular, we find that PT and physiotherapy students in Lebanon believe their role in CR is important. Several studies conducted have demonstrated the importance of the role played by the PT in the promotion of CR. PT act as a bridge between patients and other healthcare professionals, and this has been confirmed by diverse findings and has been recommended by international guidelines and the WHO [5] and is highlighted by our results. PT’s qualifications and their ability to screen, diagnose, and provide appropriate treatment or referral have positioned them as important providers of quality health care within the primary care team [8].

As described in our results, PT and physiotherapy students believe that CR has the potential to improve quality of life of patients. The efficacy of comprehensive exercise-based rehabilitation programs has been demonstrated worldwide, and these programs are considered essential in the management of CVDs [7] and pulmonary diseases [9]. CR has been shown to significantly reduce all causes of mortality [10]. CR can increase health outcomes for patients suffering from NCDs, increase psychological well-being, and support early return to work and the development of self-management skills [11–15]. Overall, CR is one of the most cost-effective treatments available for CVDs and pulmonary diseases in association with medications therapy [16].

Our results show that Lebanese PT and physiotherapy students are encouraging physical activity in their daily practice beyond their therapeutic exercises. Our study underlines a positive attitude toward the implementation of future CR center in the country. PT agree that CR centers will have an added value for the country. Our survey found that PT contribute to the promotion of a healthy life to the patient post-accident or surgery, as indicated by the participants.

According to several studies and international guidelines, physical inactivity is linked to low levels of physical fitness, and it is considered a major and independent risk factor in the development and progression for CVDs [17]. PT are playing a role in the care process by prescribing structured exercise programs as a therapeutic intervention and promoting CR in the prevention of disease complication [18–20]. The profession of physiotherapy helps millions of people every year to prevent NCDs and their risk factors [21, 22]. NCDs are collectively responsible for 71% of all deaths worldwide [23–26]. Three quarters of all NCD deaths occur in low- and middle-income countries [27].

The CR field in Lebanon is still largely absent and ranks lower than the neurological and musculoskeletal domains or fitness, which are the top physiotherapy activities. This was showed by our findings as well. This is largely due to the consequences of a long period of war and the benefits to earn more money in the country by treating first the orthopedics accidents and post-surgery patients. In their daily practice, PT in Lebanon offer very few cardiopulmonary treatments. In fact they are facing barriers due to the low level of referrals by physicians.

As shown in our findings, most of the respondents stated that patients suffering from NCDs (post cardiac event, cardiac surgery, COPD patients, or with pulmonary diseases) will be “eligible candidates” for CR. But our results showed that the number of patient treatment in the cardiopulmonary field is still very low, and home-based treatment is practically absent according to our findings. Therefore, awareness and education are needed among primary healthcare providers to overcome these barriers in the practice of PT. More powerful and enthusiastic endorsement at or after the discharge of the patients is suggested to increase CR treatments. A study suggested that a “motivational letter” provided to the patients can be helpful to remind the physicians to initiate a CR referral [28].

Our findings confirm that PT and physiotherapy students have excellent knowledge of CR and support all kinds of CR programs in Lebanon, which could reduce CVDs and pulmonary diseases [29]. They are defined as diseases of long duration, generally slow progression and are the major cause of adult mortality and morbidity worldwide. CVDs and pulmonary diseases are identified by the WHO as “Group II Diseases”, a category of diseases that aggregates based on ICD-10 code the conditions and causes of deaths for respiratory diseases (e.g., COPD, asthma, other) and CVDs [30]. Four main diseases are generally considered to be dominant in NCD mortality and morbidity: CVDs (including heart disease and stroke), diabetes, cancer, and chronic respiratory diseases (including chronic obstructive pulmonary disease and asthma) (https://www.who.int/nmh/publications/ncd-infographic-2014.pdf). These groups are linked by common risk factors like tobacco, nutrition, and physical inactivity and are modifiable risk factors that an individual can change and professional health providers could change by applying therapy, like rehabilitation to improve health outcomes of the patients (https://www.who.int/nmh/publications/ncd-infographic-2014.pdf). For that purpose, promoting CR as a cost-effective prevention is a crucial point, above all in low- and middle-income countries, like Lebanon. This can help to decrease the burden of CVDs and pulmonary diseases, which are number one according to WHO and the Ministry of Public Health [31, 32].

Finally, our results are similar to studies conducted as PT play a key role in the development and growth of CR [28]. This was confirmed in different studies in the UK [28], Australia [6], Cyprus [33], Greece, and the USA [34]. These studies confirm that the presence of CR programs is important. A study conducted by Karam Turk-Adawi [35] found that worldwide, there is low availability of CR; only 38.8% of countries globally have CR programs. Specifically, 68.0% of high-income and 23% of LMICs (28.2% for middle- and 83% for low-income countries) have CR. CR density estimates ranged from 1 program per 01–64 million inhabitants.

PT join physicians and nurses and other members of a multidisciplinary team to promote patient’s health and help them to return to their daily life activities. Other studies found that PT have a role in improving physical activity of the patient in all CR phases [36]. Overall, evidence from several studies demonstrated that preoperative and postoperative physiotherapies contribute to early functional recovery and reduce hospitalization stay and mortality rate [37, 38].

### Limitations

Our study has some limitations; the survey was done only in English and not in Arabic or French. Although we recognize this limitation, we do not expect that it had a substantial influence on the results because most respondents were able to communicate in English. Nevertheless, some of the questions might have seemed hypothetical to the respondents since CR is new in Lebanon and participants might not have known about the presence of a CR program in two medical establishments. Another limitation was the difficulty to properly give explanations of the questionnaire to every participant due to the overcrowded conference registration desk where the questionnaire was distributed. Another limitation was the lack of motivation of some conference attendance to fill in the survey questionnaire before the congress, which resulted in a somewhat lower response rate. Thus, not all Lebanese PT and physiotherapy students contacted and invited to attend the congress participated in this survey. This was especially observed among the PT students. Therefore, we cannot exclude self-selection bias. Specifically, participants in the survey might have been more interested in CR and more positive about CR than the rest of the PT and physiotherapy students who chose not to participate. This might have skewed the results toward positive opinions. In addition, we assumed that the sample was fairly representative because all Lebanese physiotherapists at the national congress of the OPTL and all Lebanese physiotherapists registered in Lebanon were invited to attend. We recognize, however, that not all contacted physiotherapists participated and that it could be a limitation.

## Conclusions

As indicated by our results and their discussion, PT are playing a major role in the promotion of CR and prevention in NCDs. This first study conducted in Lebanon highlighted the role played by the Lebanese PT who act as an active bridge in the healthcare system and can help to initiate CR programs. Additional training will be crucial for the profession. Specifically, Lebanese PT need more information and training on CR, which suggests that education programs in that area are important to be developed and offered by universities to create more and better-informed PT specialists in this field. Supplementary learning for professional healthcare providers, patients, and medical students about the benefits of CR is highly recommended to enroll more patients into rehabilitation. CR coaching needs to be initiated in this field in Lebanon, as well as in other low- and middle-income countries, above all in time of the COVID-19 pandemic. Barriers faced to enter a CR need to be diminished by covering the CR programs by the health insurance companies and the Lebanese health authorities. By ensuring that CR is available for cardiac and pulmonary patients and expenses are covered, a reduction in cardiopulmonary morbidity and mortality can be expected at a national level, which is the top chronic disease problem in Lebanon.

### Recommendation for practical implementation in the country

Our study has also explored the recommendations of respondents for the further development of CR and its practical implementation in Lebanon. The first recommendation suggested by the respondents is to have “more training and education” in CR and to raise their awareness about CR importance. Also, the first two barriers to CR indicated by our results are “the missing of skills” by the PT and “the missing of specialties” in this domain. Comprehensive training programs are not offered in the Lebanese’s universities until now. Occasionally, the OPTL have foreign lecturers to give training in CR. A strong endorsement in every medical establishment to initiate a CR treatment is needed and should be part of the patient’s discharging policy and procedure.

A second recommendation based on our findings is that there is a need to solve the issue of who covers the costs of patients’ enrollment in CR. This point needs to be considered by the Lebanese healthcare authorities by covering the rehabilitation component in prevention programs. The Ministry of Public Health in Lebanon needs to recognize the important role that PT play in the delivery of cost-effective CR [39] and in the reduction of NCD risk factors, which are primary concerns in Lebanon. The healthcare system in Lebanon is funded more through private than public resources [40]. A cost-effective CR program deserves to be covered by the private and public systems. At present, there is no funding in the country for primary care due to the instable political and economic situation. In addition, no national strategic plan is available for the implementation of CR centers, and no budget is given from any private fund or by the government concerning the CR at the time being.

The third recommendation is based on the role played by PT in the promotion of CR and prevention of NCDs, which is crucial and needs to be developed in the near future. Patients suffering from CVDs and pulmonary diseases, such as the COVID-19 survivors, need to start a CR program to get back to independent life and to work as soon as possible. The importance of the role played by the PT in the CR after COVID-19 have been already demonstrated in several studies [41–43]. Therefore, CR has an important role in Lebanon, and in other low- and middle-income countries due to its cost-effective components [44].

The final recommendation is related to access to CR. Our findings show that PT face barriers to enroll patients into CR. The location and travel distance to the rehabilitation centers was in the top 5 difficulties faced in Lebanon. At present, two CR programs have opened in two hospitals in Lebanon, one in Beirut West and another one in Mount-Lebanon area, which will facilitate the access for all kinds of patients suffering from NCDs in the Beirut district. Another barrier to the implementation of the CR program is the impact of the unstable political and economic situation of country on the Lebanese healthcare system, which was not covered by our study and need further investigation. As reported by international and local sources [45], at present, the medical suppliers in Lebanon who provide equipment for both public and private hospitals account for 82% of Lebanon healthcare capacity. The Lebanese healthcare system is unable to provide and import medical equipment due to the shortage of financial resources and the absence of government regulations that prevent banks from arbitrarily restricting money [40–45].

## Supplementary Information

**Additional file 1.** Survey questionnaire

**Additional file 2.** Sociodemographic characteristics of the respondents

**Additional file 3.** Tables

## Data Availability

Data are stored in Maastricht University and are available upon request.

## Abbreviations

BMI: Body mass index
CR: Cardiopulmonary rehabilitation
COPD: Chronic obstructive pulmonary diseases
CVDs: Cardiovascular diseases
KAP: Knowledge, attitudes and practices
NCDs: Non-communicable diseases
NSSF: National Social Security Fund
OPTL: Ordre des Physiothérapeutes du Liban
PT: Physiotherapists
WHO: World Health Organization
WCPT: American Physical Therapy Association and World Confederation of Physical Therapy
