# Supplementary material for: Knowledge, attitudes, and practices of cardiopulmonary rehabilitation among physiotherapists in Lebanon
Source: Bull Fac Phys Ther. 2022 Jan 12;27(1):2. doi: 10.1186/s43161-021-00060-w (PMC8752176; doi:10.1186/s43161-021-00060-w)
Supplement: Supplementary file 1 — Additional file 1. Survey questionnaire [file 43161_2021_60_MOESM1_ESM.docx]

**Additional file 1: Survey questionnaire**

| SURVEY  **This survey aims to analyze the attitude, knowledge and practice of physiotherapists regarding secondary prevention and promotion of cardiopulmonary rehabilitation (CPR) This survey is anonymous, and data will be used for research purposes only We would highly appreciate your cooperation We highlight the importance of your collaboration for the future of rehabilitation in the country Please fill out this questionnaire and place it in the box in the lobby Thank you!**  **Do you agree to participate?** ◻  **Part1:**  1 What is your level of knowledge about cardiopulmonary rehabilitation (CPR)? Please rate 1-5  ◻ 1 ◻ 2 ◻ 3 ◻ 4 ◻ 5  Very poor Excellent  2 What is your level of knowledge about the multidisciplinary components of CPR? Please rate 1-5  ◻ 1 ◻ 2 ◻ 3 ◻ 4 ◻ 5  Very poor Excellent  3 What is your level of knowledge about WHO and WCPT guidelines for healthy promotion for CPR? Please rate 1-5  ◻ 1 ◻ 2 ◻ 3 ◻ 4 ◻ 5  Very poor Excellent  4 Do you adhere to international guidelines in promoting recommendations for non-communicable diseases in secondary prevention?  ◻ 1 ◻ 2 ◻ 3 ◻ 4 ◻ 5  Very poor Excellent  **Part 2:**  1 What kind of patients do you consider suitable for CPR after discharging from hospital? Multiples answers are possible:  ◻ Post cardiac event or surgery  ◻ COPD  ◻ Post any kind of surgery  ◻ Muscular dystrophy  ◻ Cancer survivors  ◻ Diabetics  ◻ Pulmonary diseases  ◻ Before & after Transplantation surgery  ◻Obese |  | 2 Do you think it would be difficult for a PT to refer patients to CPR in the country?  ◻ Yes \| ◻ No  3Who should take initiative to initiate this kind of program in the country? Multiples answers are possible:  ◻ Insurance companies  ◻ Physical Therapists  ◻ Physicians  ◻ Policy providers  4How many patients did you treat suffering from cardiopulmonary diseases during last month at your work place?  ◻ 0 ◻ 1-2 ◻ 3-10 ◻ 11-20 ◻ >21  5How many patients did you treat suffering from cardiopulmonary diseases during last month at patients ‘home?  ◻ 0 ◻ 1-2 ◻ 3-10 ◻ 11-20 ◻ >21  **Part 3:**  1 Do you observe any barriers when patients are referred from Physicians/ Primary care providers to start a Rehabilitation program? ◻ Yes \| ◻ No  2 If yes, what kind of barrier(s) do you faced?  ◻ More skills are needed in Lebanon  ◻ More specialists are needed in Lebanon  ◻ More equipped centers are needed in Lebanon  ◻ Lack of interest in Cardiopulmonary Rehab  ◻ CPR would be not beneficial for the patient  ◻ CPR would not change the patient behavior  ◻ Price of the program  ◻ Not covering by insurance and NSSF  ◻ No enough endorsement by physicians to start CPR program  ◻ Others:  **Part 4:** Do you agree that the role of the physiotherapist is:  -To discuss the benefits of a healthy and active lifestyle with the patient  ◻ agree \| ◻ disagree  -To promote the prevention of cardiovascular diseases ◻ agree \| ◻ disagree  -To encourage Physical activity in their clinical practice on a daily basic (beyond therapeutic exercises)  ◻ agree \| ◻ disagree |
| --- | --- | --- |

| -To be confident when giving advices to the patient on physical activity?  ◻ agree \| ◻ disagree  -To be active, healthy to act as a model for their patients ◻ agree \| ◻ disagree  -To assess patients: ie: BMI, make screening of Cardiovascular risk factors ◻ agree \| ◻ disagree  -To Prescribe exercise counselling according to the patient assessment beyond exercises therapy ◻ agree \| ◻ disagree  -To assess exercises capacity ie: via 6-minute walk test ◻ agree \| ◻ disagree  -To give diet/ Nutritional counselling  ◻ agree \| ◻ disagree  -To give smoking cessation counselling ◻ agree \| ◻ disagree  -To make Patient education to help them cope with their illness and improve their health-related quality of life ◻ agree \| ◻ disagree  -Tno give psychological management during treatment ◻ agree \| ◻ disagree  -To make recommendations post treatment on exercises ◻ agree \| ◻ disagree  **Part 5**:  1 Do you agree that cardiopulmonary rehabilitation in Lebanon is effective? ◻ agree \| ◻ disagree  2Do you agree that access for an outpatient Center is an added value in the country? ◻ agree \| ◻ disagree  3Do you agree that CPR could improve the quality of life and lifestyle of a stable patient post-surgery or suffering from cardiovascular & pulmonary diseases?  ◻ agree \| ◻ disagree  4Do you agree that CPR could change patient behaviors post-surgery or suffering from cardiovascular diseases?  ◻ agree \| ◻ disagree  **Part 6:**  1Do you support outpatient CPR? ◻ Yes \| ◻ No  2Do you support inpatient CPR? ◻ Yes \| ◻ No  3Do you support home-based Tele rehabilitation?  ◻ Yes \| ◻ No |  | **Part 7:**  Age:  ◻ <25 ◻ 26-35 ◻ 36-45 ◻ 46-55 ◻ 56-65 ◻ >65  Gender:  ◻ F \| ◻ M  Level of education:  ◻ PT student ◻ BSc PT  ◻ MSc PT ◻ Others Master’s degree  ◻ DPT ◻ PhD  ◻ Others (please specify): ______________________  Place of working:  ◻ Beirut ◻ Mont Lebanon  ◻ South Lebanon ◻ North Lebanon  ◻ Beqaa  Number of years of practice:  ◻ 1-10 ◻ 11-20 ◻ 21-30 ◻ 31-45 ◻ >45  Main area of practice (please tick your *major* area):  ◻ General practice in PT  ◻ Musculo-squeletic ◻ Neurology  ◻ Pediatric ◻ Cardiopulmonary  ◻ Beauty/Spa ◻ Sport Medicine  ◻ Geriatric ◻ Manual Therapy  Number of patients treated per week:  ◻ 1-10 ◻ 11-20 ◻ 21-30 ◻ 31-45 ◻ >45  Working hours per week  ◻ 1-10 ◻ 11-20 ◻ 21-30 ◻ 31-45 ◻ >45  Workplace setting:  ◻ Inpatient Hospital setting  ◻ Outpatient Hospital setting  ◻ Private clinic  ◻ Education/ university  How do you rate your lifestyle behavior?  ◻ 1 ◻ 2 ◻ 3 ◻ 4 ◻ 5  Very poor Excellent  **Thank you!** |
| --- | --- | --- |
