# Supplementary material for: Knowledge, attitudes, and practices of cardiopulmonary rehabilitation among physiotherapists in Lebanon
Source: Bull Fac Phys Ther. 2022 Jan 12;27(1):2. doi: 10.1186/s43161-021-00060-w (PMC8752176; doi:10.1186/s43161-021-00060-w)

**Additional file 2: Socio-demographic characteristics of the respondents**

Figure 1: Age of respondents


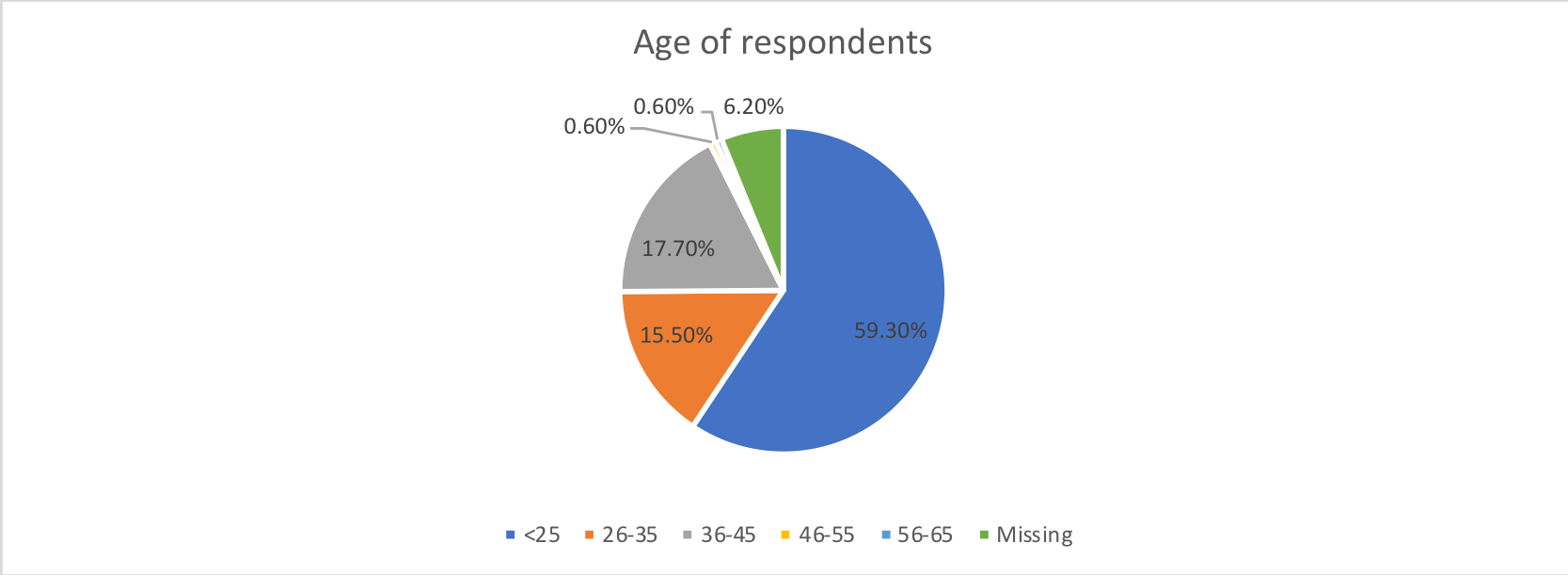


Figure 2: Gender of respondents


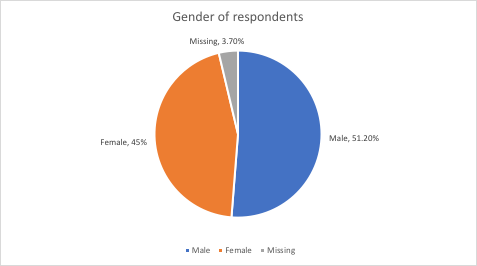


Figure 3: Education level of the respondents


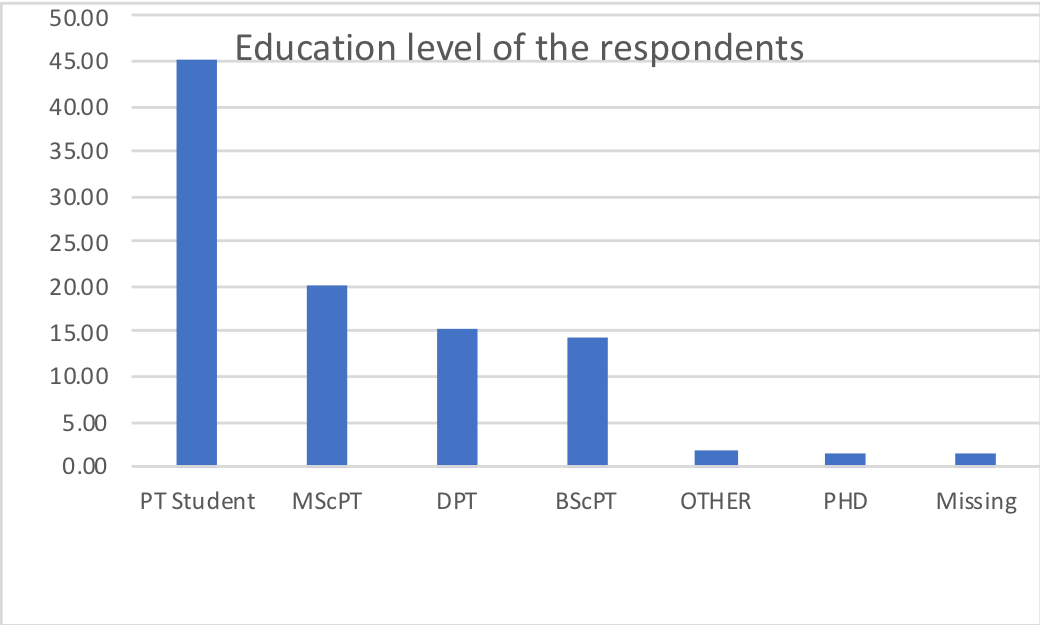


Figure 4: Work’s categories of the respondents


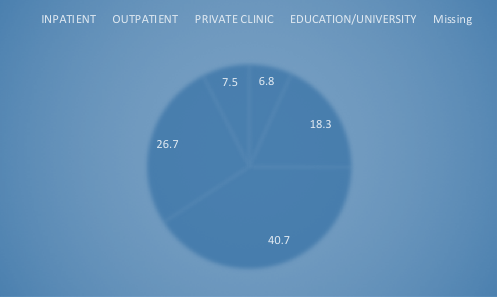


Figure 5: Home place of the respondents


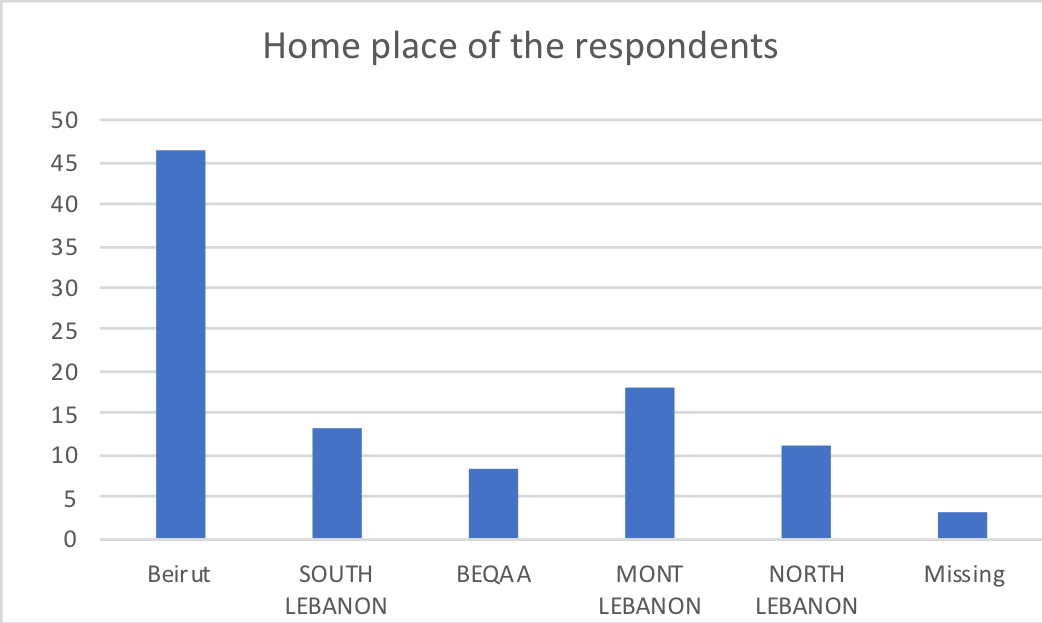

Supplement: Supplementary file 2 — Additional file 2. Sociodemographic characteristics of the respondents [file 43161_2021_60_MOESM2_ESM.docx]
