# Supplementary material for: Knowledge, attitudes, and practices of cardiopulmonary rehabilitation among physiotherapists in Lebanon
Source: Bull Fac Phys Ther. 2022 Jan 12;27(1):2. doi: 10.1186/s43161-021-00060-w (PMC8752176; doi:10.1186/s43161-021-00060-w)
Supplement: Supplementary file 3 — Additional file 3. Tables [file 43161_2021_60_MOESM3_ESM.docx]

**Additional file 3: Tables**

Table 1: Knowledge of physiotherapists and physiotherapists students in Lebanon about cardiopulmonary rehabilitation

| **Knowledge** |  | **Total sample**  **N=322**  **n (%)** | **Physiotherapists**  **N=176**  **n (%)** | **Physiotherapist students**  **N=146**  **n (%)** |
| --- | --- | --- | --- | --- |
| **What is your level of knowledge about cardiopulmonary rehabilitation?** |  |  |  |  |
|  | **Very poor** | 28(8.7%) | 12(6.8%) | 16(11.0%) |
|  | **Poor** | 69(21.4%) | 12(6.8%) | 57(39.0%) |
|  | **Medium** | 133(41.3%) | 79(44.9%) | 54(37.0%) |
|  | **Good** | 65(20.2%) | 52(29.5%) | 13(8.9%) |
|  | **Excellent** | 14(4.3%) | 13(7.4%) | 1(0.7%) |
|  | **n (missing**) | 13(4.0%) | 8(4.5%) | 5(3.4%) |
| **What is your level of knowledge about the multidisciplinary components of cardiopulmonary rehabilitation?** |  |  |  |  |
|  | **Very poor** | 22(6.8%) | 17(9.7%) | 5(3.4%) |
|  | **Poor** | 65(20.2%) | 10(5.7%) | 55(37.7%) |
|  | **Medium** | 145(45.0%) | 61(34.7%) | 84(57.5%) |
|  | **Good** | 76(23.6%) | 75(42.6%) | 1(0.7%) |
|  | **Excellent** | 6(1.9%) | 5(2.8%) | 1(0.7%) |
|  | **n (missing)** | 8(2.5%) | 8(4.5%) | 0(0%) |
| **What is your level of knowledge about WHO and WCPT guidelines for healthy promotion for cardiopulmonary rehabilitation** |  |  |  |  |
|  | **Very poor** | 29(9.0%) | 17(9.7%) | 12(8.2%) |
|  | **Poor** | 53(16.5%) | 28(15.9%) | 25(17.1%) |
|  | **Medium** | 83(25.8%) | 51(29.0%) | 32(21.9%) |
|  | **Good** | 137(42.5%) | 63(35.8%) | 74(50.7%) |
|  | **Excellent** | 11(3.4%) | 9(5.1%) | 2(1.4%) |
|  | **n (missing)** | 9(2.8%) | 8(4.5%) | 1(0.7%) |
| **Do you adhere to international guidelines in promoting recommendations for non-communicable diseases in secondary prevention?** |  |  |  |  |
|  | **Very poor** | 48(14.9%) | 26(14.8%) | 22(15.1%) |
|  | **Poor** | 31(9.6%) | 20(11.4%) | 11(7.5%) |
|  | **Medium** | 66(20.5%) | 47(26.7%) | 19(13.0%) |
|  | **Good** | 148(46.0%) | 64(36.4%) | 84(57.5%) |
|  | **Excellent** | 20(6.2%) | 11(6.3%) | 9(6.2%) |
|  | **n (missing)** | 9(2.8%) | 8(4.5%) | 1(0.7%) |
|  |  |  |  |  |
| **Total** | **N (%)** | 322(100%) | 176(100%) | 146(100%) |

Table 2: Attitude of physiotherapists and physiotherapists students towards cardiopulmonary rehabilitation

| **Attitude** |  | **Total sample**  **N=322**  **n (%)** | **Physiotherapists**  **N=176**  **n (%)** | **Physiotherapist**  **Students**  **N=146**  **n (%)** |
| --- | --- | --- | --- | --- |
| **Do you agree that cardiopulmonary rehabilitation in Lebanon is effective?** |  |  |  |  |
|  | **Agree** | 115(35.7%) | 68(38.6%) | 47(32.2%) |
|  | **Disagree** | 196(60.9%) | 101(57.4%) | 95(65.1%) |
|  | **n (missing)** | 11(3.4%) | 7(4.0%) | 4(2.7%) |
| **Do you agree that access for an outpatient canter is an added value in in Lebanon?** |  |  |  |  |
|  | **Agree** | 306(95.0%) | 168(95.5%) | 138(94.5%) |
|  | **Disagree** | 2(0.6%) | 1(0.6%) | 1(0.7%) |
|  | **n (missing)** | 14(4.3%) | 7(4.0%) | 7(4.8%) |
| **Do you agree that cardiopulmonary rehabilitation could increase quality of life and lifestyle of the patient?** |  |  |  |  |
|  | **Agree** | 308(95.7%) | 168(95.5%) | 140(95.9%) |
|  | **Disagree** | 2(0.6%) | 1(0.6%) | 1(0.7%) |
|  | **n (missing)** | 12(3.7%) | 7(4.0%) | 5(3.4%) |
| **Do you agree that cardiopulmonary rehabilitation could change patient behaviors post-surgery or suffering from cardiovascular diseases?** |  |  |  |  |
|  | **Agree** | 307(95.3%) | 168(95.5%) | 139(95.2%) |
|  | **Disagree** | 2(0.6%) | 1(0.6%) | 1(0.7%) |
|  | **n (missing)** | 13(4.0%) | 7(4.0%) | 6(4.1%) |
|  |  |  |  |  |
| **Total** | **N (%)** | 322(100%) | 176(100%) | 146(100%) |

Table 3.1: Practice of physiotherapists and physiotherapists students in Lebanon regarding cardiopulmonary rehabilitation

| **Practice** |  | **Total sample**  **N=322**  **n(%)** | **Physiotherapists**  **N=176**  **n(%)** | **Physiotherapists students**  **N=146**  **n(%)** |
| --- | --- | --- | --- | --- |
| **What kind of patients do you consider suitable for cardiopulmonary rehabilitation after discharging from hospital?** |  |  |  |  |
| **Cardiac patients** | **Yes** | 237(73.6%) | 132(75%) | 105(71.9%) |
|  | **No** | 79(24.5%) | 40(22.7%) | 39(26.7%) |
|  | **n(missing)** | 6(1.9%) | 4(2.3%) | 2(1.4%) |
|  |  |  |  |  |
| **COPD patients** | **Yes** | 271(84.1%) | 142(80.7%) | 129(88.3%) |
|  | **No** | 45(14%) | 30(17.0%) | 15(10.3%) |
|  | **n(missing)** | 6(1.9%) | 4(2.3%) | 2(1.4%) |
|  |  |  |  |  |
| **Post-surgery patients** | **Yes** | 126(39.1%) | 71(40.3%) | 55(37.6%) |
|  | **No** | 190(59%) | 101(57.4%) | 89(61%) |
|  | **n(missing)** | 6(1.9%) | 4(2.3%) | 2(1.4%) |
|  |  |  |  |  |
| **Muscular dystrophy patients** | **Yes** | 83(25.7%) | 50(28.4%) | 33(22.6%) |
|  | **No** | 233(72.3%) | 122(69.3%) | 111(76%) |
|  | **n(missing)** | 6(1.9%) | 4(2.3%) | 2(1.4%) |
|  |  |  |  |  |
| **Cancer patients** | **Yes** | 72(22.96%) | 45(25.6%) | 27(18%) |
|  | **No** | 244(77.83%) | 127(72.2%) | 117(78%) |
|  | **n(missing)** | 6(1.9%) | 4(2.3%) | 2(1.4%) |
|  |  |  |  |  |
| **Diabetic patients** | **Yes** | 70(21.73%) | 43(24.4%) | 27(18.5%) |
|  | **No** | 246(76.4%) | 129(73.3%) | 117(80.1%) |
|  | **n(missing)** | 6(1.9%) | 4(2.3%) | 2(1.4%) |
|  |  |  |  |  |
| **Patients suffering from pulmonary diseases** | **Yes** | 248(77.1%) | 135(76.7%) | 113(77.4%) |
|  | **No** | 68(21.1%) | 37(21.0%) | 31(21.2%) |
|  | **n(missing)** | 6(1.9%) | 4(2.3%) | 2(1.4%) |
|  |  |  |  |  |
| **Transplantation patients** | **Yes** | 157(48.7%) | 88(50%) | 69(47.2%) |
|  | **No** | 159(49.4%) | 84(47.7%) | 75(51.4%) |
|  | **n(missing)** | 6(1.9%) | 4(2.3%) | 2(1.4%) |
|  |  |  |  |  |
| **Obese patients** | **Yes** | 57(17.7%) | 42(23.9%) | 15(10.3%) |
|  | **No** | 259(80.4%) | 130(73.9%) | 129(88.3%) |
|  | **n(missing)** | 6(1.9%) | 4(2.3%) | 2(1.4%) |
|  |  |  |  |  |
| **Total** | **N(%)** | 322(100%) | 176(100%) | 146(100%) |

Table 3.2: Practice of physiotherapists and physiotherapists students in Lebanon regarding cardiopulmonary rehabilitation

| **Practice** |  | **Total sample**  **N=322**  **n (%)** | **Physiotherapists**  **N=176**  **n (%)** | **Physiotherapists**  **Student**  **N=146**  **n (%)** |
| --- | --- | --- | --- | --- |
| **Do you think it would be difficult for a physiotherapist to refer patients to CR in in Lebanon?** |  |  |  |  |
|  | **Yes** | 250(77.6%) | 141(80.1%) | 109(74.7%) |
|  | **No** | 66(20.5%) | 31(17.6%) | 35(23.97%) |
|  | **n (missing)** | 6(1.9%) | 4(2.3%) | 2(1.36%) |
| **Who should take initiative to initiate cardiopulmonary rehabilitation in in Lebanon?** |  |  |  |  |
| **Insurance companies** | **Yes** | 131(40.7%) | 71(40.3%) | 60(40%) |
|  | **No** | 185(57.5%) | 101(57%) | 84(56%) |
|  | **n (missing)** | 6(1.9%) | 4(2.3%) | 2(1.4%) |
|  |  |  |  |  |
| **Physiotherapists** | **Yes** | 231(71.7%) | 128(72%) | 103(70.5%) |
|  | **No** | 85(26.4%) | 44(25.7%) | 41(28.1%) |
|  | **n (missing)** | 6(1.9%) | 4(2.3%) | 2(1.4%) |
|  |  |  |  |  |
| **Doctors** | **Yes** | 218(67.7%) | 118(67%) | 100(66.7%) |
|  | **No** | 98(30.4%) | 54(30%) | 44(29.3%) |
|  | **n (missing)** | 6(1.9%) | 4(2.3%) | 2(1.4%) |
|  |  |  |  |  |
| **Policy providers** | **Yes** | 170(52.8%) | 84(47%) | 86(58.9) |
|  | **No** | 146(45.3%) | 88(50.7%) | 58(39.7%) |
|  | **n (missing)** | 6(1.9%) | 4(2.3%) | 2(1.4%) |
|  |  |  |  |  |
| **Total** | **N  (%)** | 322(100%) | 176(100%) | 146(100%) |

Table 3.3: Practice of physiotherapists and physiotherapists students in Lebanon regarding cardiopulmonary rehabilitation

| **Practice** |  | **Total sample**  **N=322**  **n (%)** | **Physiotherapists**  **N=176**  **n (%)** | **Physiotherapists**  **Students**  **N=146n (%)** |
| --- | --- | --- | --- | --- |
| **How many patients did you treat suffering from cardiopulmonary diseases during last month at your work place?** |  |  |  |  |
|  | **0** | 272(84.5%) | 149(84.7%) | 123(82%) |
|  | **1-2** | 26(8.1%) | 11(6.3%) | 15(10.2%) |
|  | **3-10** | 16(5.0%) | 10(5.7%) | 6(4%) |
|  | **11-20** | 0(0%) | 2(1.1%) | 0(0%) |
|  | **>21** | 0(0%) | 0(0%) | 0(0%) |
|  | **n (missing)** | 8(2.4%) | 4(2.2%) | 4(2.8%) |
| **How many patients did you treat suffering from cardiopulmonary diseases during last month at patients ‘home?** |  |  |  |  |
|  | **0** | 285(88.5%) | 156(88.6%) | 129(88.35%) |
|  | **1-2** | 17(5.3%) | 8(4.5%) | 9(6%) |
|  | **3-10** | 12(3.7%) | 6(3.4%) | 6(4%) |
|  | **11-20** | 1(0.3%) | 1(0.6%) | 0(0%) |
|  | **>21** | 1(0.3%) | 0(0%) | 1(0.7%) |
|  | **n (missing)** | 6(1.9%) | 5(2.9%) | 1(0.7%) |
|  |  |  |  |  |
| **Total** | **N (%)** | 322(100%) | 176(100%) | 146(100%) |

Table 4: Barriers faced by physiotherapists and physiotherapists students to refer patients to cardiopulmonary rehabilitation in Lebanon

| **Barriers** |  | **Total sample**  **N=322**  **n (%)** | | **Physiotherapists**  **N=176**  **n (%)** | | **Physiotherapists students**  **N=146n (%)** |
| --- | --- | --- | --- | --- | --- | --- |
| **Do you observe any barriers when patients are referred from physicians/ primary care providers to start a rehabilitation program?** |  |  | |  | |  |
|  | **Yes** | 207(64.3%) | | 115(65.3%) | | 92(61.3%) |
|  | **No** | 87(27.0%) | | 43(24.5%) | | 44(29.3%) |
|  | **n (missing)** | 28(8.7%) | | 18(10.2%) | | 10(7.4%) |
| **What kind of barriers do you faced?** |  |  | |  | |  |
| **More skills are needed in Lebanon** |  |  | |  | |  |
|  | **Yes** | 177(54.97%) | | 99(56.3%) | | 78(53.42%) |
|  | **No** | 119(37%) | | 56(31.8%) | | 63(43.15%) |
|  | **n (missing)** | 26(8.07%) | | 21(11.9%) | | 5(3.42%) |
| **More specialists are needed in Lebanon** |  |  |  | |  | |
|  | **Yes** | 127(39.4%) | 76(43.2%) | | 51(34.93%) | |
|  | **No** | 169(52.5%) | 79(44.9%) | | 90(61.64%) | |
|  | **n (missing)** | 26(8.1%) | 21(11.9%) | | 5(3.42%) | |
| **More equipped centers are needed in Lebanon** |  |  |  | |  | |
|  | **Yes** | 100(31.1%) | 55(31.2%) | | 45(30%) | |
|  | **No** | 198(61.5%) | 102(58.0%) | | 96(64%) | |
|  | **n (missing)** | 24(7.4%) | 19(10.8%) | | 5(3.6%) | |
| **Lack of interest in cardiopulmonary rehabilitation** |  |  |  | |  | |
|  | **Yes** | 70(21.7%) | 25(14.2%) | | 45(31.5%) | |
|  | **No** | 226(70.2%) | 130(73.86%) | | 96(65%) | |
|  | **n (missing)** | 26(8.07%) | 21(11.93%) | | 5(3.5%) | |
| **Cardiopulmonary rehabilitation would benefit the patient** |  |  |  | |  | |
|  | **Yes** | 52(16.1) | 26(14.8%) | | 26(17.8%) | |
|  | **No** | 244(75.8%) | 129(73.3%) | | 115(78.7%) | |
|  | **n (missing)** | 26(8.07%) | 21(11.9%) | | 5(3.4%) | |
| **Cardiopulmonary rehabilitation would not change the patient behavior** |  |  |  | |  | |
|  | **Yes** | 52(16.1) | 26(14.8%) | | 26(17.8%) | |
|  | **No** | 244(75.8%) | 129(73.3%) | | 115(78.76%) | |
|  | **n (missing)** | 26(8.1%) | 21(11.9%) | | 5(3.42%) | |
| **Price of the cardiopulmonary rehabilitation program will be a barrier** |  |  |  | |  | |
|  | **Yes** | 127(39.4%) | 76(43.2%) | | 51(34.9%) | |
|  | **No** | 169(52.5%) | 79(44.9%) | | 90(61.6%) | |
|  | **n (missing)** | 26(8.1%) | 21(11.9%) | | 5(3.5%) | |
| **Not covering by insurances companies and NSSF will be a barrier** |  |  |  | |  | |
|  | **Yes** | 100(31.1%) | 55(31.3%) | | 45(30.8%) | |
|  | **No** | 198(61.5%) | 102(58.0%) | | 96(65.7%) | |
|  | **n (missing)** | 24(7.45%) | 19(10.8%) | | 5(3.42%) | |
| **Not enough endorsement by physicians will be a barrier** |  |  |  | |  | |
|  | **Yes** | 109(33.9%) | 42(23.9%) | | 46(31.5%) | |
|  | **No** | 189(58.7%) | 115(65.3%) | | 95(100%) | |
|  | **n (missing)** | 24(7.45%) | 19(10.8%) | | 5(3.5%) | |
|  |  |  |  | |  | |
| **Others kind of barriers:** | **Yes** | 79(24.5%) | 57(32.4%) | | 22(15.06%) | |
|  | **No** | 217(67.4%) | 100(56.8%) | | 117(80.1%) | |
|  | **n (missing)** | 26(8.07%) | 19(10.8%) | | 7(4.79%) | |
|  |  |  |  | |  | |
| **Total** | **N (%)** | 322(100%) | 176(100%) | | 146(100%) | |

Table 5: Role played by physiotherapists and physiotherapists students in the promotion of cardiopulmonary rehabilitation in Lebanon

| **Role of physiotherapist** |  | **Total sample**  **N=322**  **n (%)** | **Physiotherapists**  **N=176**  **n (%)** | **Physiotherapists student**  **N=146n (%)** |
| --- | --- | --- | --- | --- |
| **Role of physiotherapist is:** |  |  |  |  |
| **To discuss the benefits of a healthy and active lifestyle with the patients** |  |  |  |  |
|  | **Agree** | 310(96.3%) | 170(96.6%) | 140(95.89%) |
|  | **Disagree** | 0 (0%) | 0 (0%) | 0(0%) |
|  | **n (missing)** | 12(3.7%) | 6(3.4%) | 6(4.1%) |
| **To promote the prevention of cardiovascular diseases** |  |  |  |  |
|  | **Agree** | 310(96.3%) | 170(96.6%) | 140(95.89%) |
|  | **Disagree** | 0(0%) | 0(0%) | 0(0%) |
|  | **n (missing)** | 12(3.7%) | 6(3.4%) | 6(4.10%) |
| **To encourage physical activity in their clinical practice on a daily basic** |  |  |  |  |
|  | **Agree** | 306(95%) | 169(96%) | 137(93.83%) |
|  | **Disagree** | 2(0.6%) | 1(0.6%) | 1(0.68%) |
|  | **n (missing)** | 14(4.3%) | 6(3.4%) | 8(5.5%) |
| **To be confident when giving advices to the patient on physical activity** |  |  |  |  |
|  | **Agree** | 307(95.3%) | 161(91.5%) | 146(102.2%) |
|  | **Disagree** | 9(2.9%) | 9(5.1%) | 0(0%) |
|  | **n (missing)** | 6(1.8%) | 6(3.4%) | 0(0%) |
| **To be active, healthy to act as a model for their patients** |  |  |  |  |
|  | **Agree** | 293(91.0%) | 151(85.8%) | 132(90.4%) |
|  | **Disagree** | 18(5.6%) | 19(10.8%) | 9(6.1%) |
|  | **n (missing)** | 11(3.4%) | 6(3.4%) | 5(3.5%) |
| **To assess patients, i.e. BMI, make screening of Cardiovascular risk factors** |  |  |  |  |
|  | **Agree** | 279(86.6%) | 165(93.8%) | 114(78.08%) |
|  | **Disagree** | 28(8.7%) | 5(2.8%) | 23(15.75%) |
|  | **n (missing)** | 15(4.7%) | 6(3.4%) | 9(6.16%) |
| **To prescribe exercise counselling according to the patient assessment beyond exercises therapy** |  |  |  |  |
|  | **Agree** | 300(93.2%) | 95(54.0%) | 22(15%) |
|  | **Disagree** | 10(3.1%) | 75(42.6%) | 72(49.31%) |
|  | **n (missing)** | 12(3.7%) | 6(3.4%) | 6(4.10%) |
| **To assess exercises capacity, i.e. via 6-minute walk test** |  |  |  |  |
|  | **Agree** | 158(49.1%) | 104(59.1%) | 54(36.98%) |
|  | **Disagree** | 147(45.7%) | 57(32.4%) | 90(61.64%) |
|  | **n (missing)** | 17(5.3%) | 15(8.5%) | 2(1.4%) |
| **To give diet/ nutritional counselling** |  |  |  |  |
|  | **Agree** | 204(63.4%) | 98(55.7%) | 106(72.60%) |
|  | **Disagree** | 90(28.0%) | 72(40.9%) | 18(12.32%) |
|  | **n (missing)** | 28(8.7%) | 6(3.4%) | 22(15.06%) |
| **To give smoking cessation counselling** |  |  |  |  |
|  | **Agree** | 180(55.9%) | 98(55.7%) | 82(56.16%) |
|  | **Disagree** | 129(40.1%) | 72(40.9%) | 57(39.04%) |
|  | **n (missing)** | 13(4%) | 6(3.4%) | 7(4.8%) |
| **To make patient education to help them cope with their illness and improve their health-related quality of life** |  |  |  |  |
|  | **Agree** | 305(94.7%) | 167(94.9%) | 138(94.52%) |
|  | **Disagree** | 6(1.9%) | 3(1.7%) | 3(2.05%) |
|  | **n (missing)** | 11(3.4%) | 6(3.4%) | 5(3.43%) |
| **To give psychological management during treatment** |  |  |  |  |
|  | **Agree** | 181(56.2%) | 85(48.3%) | 96(65.75%) |
|  | **Disagree** | 129(40.1%) | 85(48.3%) | 44(30.13%) |
|  | **n (missing)** | 12(3.7%) | 6(3.4%) | 6(4.12%) |
| **To make recommendations post treatment on exercises** |  |  |  |  |
|  | **Agree** | 308(95.7%) | 169(96%) | 139(95.2%) |
|  | **Disagree** | 1(0.3%) | 1(0.6%) | 0(0%) |
|  | **n (missing)** | 13(4%) | 6(3.4%) | 7(4.8%) |
|  |  |  |  |  |
| **Total** | **N (%)** | 322(100%) | 176(100%) | 146(100%) |

Table 6: Support from physiotherapists and physiotherapists students in Lebanon to different kinds of forms of cardiopulmonary rehabilitation

| **Support** |  | **Total sample**  **N=322**  **n (%)** | **Physiotherapists**  **N = 176**  **n (%)** | **Physiotherapist students**  **N = 146n (%)** |
| --- | --- | --- | --- | --- |
| **Support to cardiopulmonary rehabilitation:** |  |  |  |  |
| **Support for inpatients cardiopulmonary rehabilitation** |  |  |  |  |
|  | **Yes** | 292(90.7%) | 163(92.6%) | 129(88.3%) |
|  | **No** | 21(6.6%) | 13(7.4%) | 8(5.5%) |
|  | **n (missing)** | 9(2.7%) | 0 | 9(6.2%) |
| **Support for outpatient cardiopulmonary rehabilitation** |  |  |  |  |
|  | **Yes** | 277(86%) | 165(93.75%) | 112(76.71%) |
|  | **No** | 21(6.5%) | 6(3.45%) | 15(10.27%) |
|  | **n (missing)** | 24(7.5%) | 5(2.8%) | 19(13.02%) |
| **Support for home-based cardiopulmonary tele-rehabilitation** |  |  |  |  |
|  | **Yes** | 241(74.8%) | 128(72.7%) | 113(77.4%) |
|  | **No** | 66(20.5%) | 41(23.3%) | 25(17.11%) |
|  | **n (missing)** | 15(4.7%) | 7(4.0%) | 8(5.5%) |
|  |  |  |  |  |
| **Total** | **N (%)** | 322(100%) | 176(100%) | 146(100%) |
